# Bacteremia Due to Lactococcus lactis cremoris Following Food Poisoning: A Case Report

**DOI:** 10.7759/cureus.66853

**Published:** 2024-08-14

**Authors:** Srujana Prabhala, Varun Khullar, Rajendra Gudisa, Arun K Sharma

**Affiliations:** 1 Microbiology, Maharishi Markandeshwar Institute of Medical Sciences and Research, Ambala, IND; 2 Neurosurgery, Maharishi Markandeshwar Institute of Medical Sciences and Research, Ambala, IND

**Keywords:** bacteremia, infectious disease, food poisoning, lactococcus

## Abstract

*Lactococcus lactis *(*L. lactis*)* cremoris* is a catalase-negative, gram-positive cocci, rarely reported as a cause of human infections. We report a case of probable food poisoning caused by *L. lactis cremoris* in an adult female. A 58-year-old female was brought to the Emergency Department with a history of sudden onset of high-grade fever, vomiting, and febrile seizures. On investigation, all parameters were within normal range. However, *L. lactis cremoris* was isolated from her blood culture. The patient gave a history of travel to a local pilgrimage and consumption of unpasteurized dairy products for a week leading up to the incident. The patient was treated with intravenous doxycycline and recovered after seven days of treatment with sterile blood cultures on follow-up.

## Introduction

*Lactococcus lactis* (*L. lactis*) is a gram-positive cocci, previously known as *Streptococcus lactis* [1]. The two main subspecies of *L. lactis* are *lactis* and *cremoris* [2]. It is widely used for the production of fermented products in the dairy industry [3]. It is generally considered to be non-pathogenic to humans. However, in some rare cases, it has been reported to cause infections such as liver abscesses, endocarditis, peritonitis, and intra-abdominal infections [4]. Since it is a skin commensal in cattle, it is thought to contaminate milk. Therefore, consumption of unpasteurized dairy products or raw milk has been reported as a common source of infection [5]. We report herein a case of food poisoning caused by *L. lactis cremoris* in an immunocompetent 58-year-old female.

## Case presentation

A 58-year-old female patient presented to the Emergency Department in an ambulatory state with complaints of fever (38.1°C), vomiting, seizures, and loss of consciousness. Routine investigations were done at the hospital, as shown in Table 1.

**Table 1:** Routine investigations

Investigations	Results
CBC	Hb: 10.2 g/dL; WBC: 14.7 x 10^3/cumm; platelet: 30 x 10^3/cumm
Urine routine microscopy and culture	In routine, all parameters were normal and culture showed no growth after overnight incubation
Stool culture	No pathogen isolated
Pro calcitonin (normal range: 0.05-0.10 ng/mL)	19.2 ng/mL
Anti-hepatitis A virus (HAV) IgM and anti-hepatitis E virus (HEV) IgM	Negative
Malaria, typhoid dot IgM, IgG dengue NS1, IgM *Leptospira*, scrub typhus IgM	Negative

**Figure 1:**
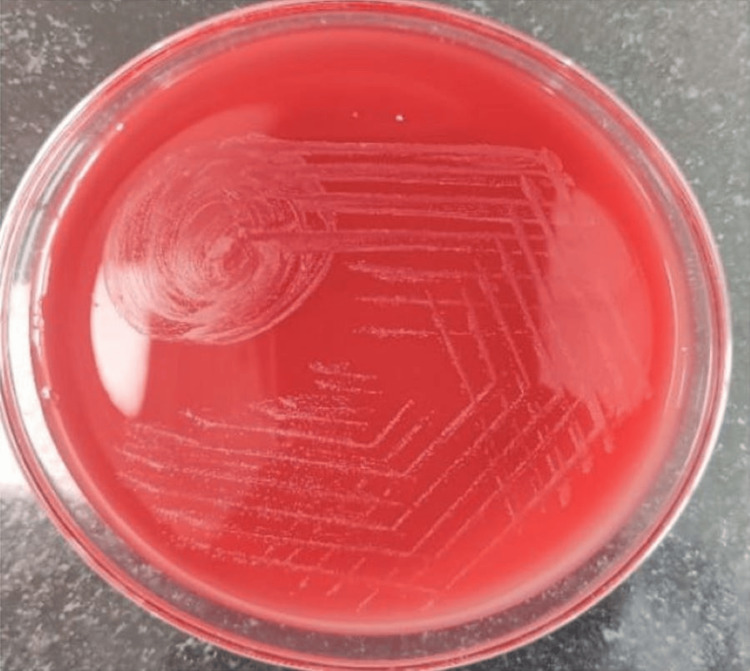
Growth of Lactococcus cremoris on blood agar

Based on the clinical presentation, an array of investigations was sent for typhoid, malaria, dengue, scrub typhus, and *Leptospira*. Two sets of blood cultures were also sent, which were incubated in the BD BACTEC FX40 blood culture system (Becton Dickinson, Franklin Lakes, NJ, USA). Intravenous (IV) ceftriaxone (2 g BD) was started as empirical treatment in view of high-grade fever. With no improvement in the clinical condition, antibiotics were escalated to IV meropenem (1 g BD) and IV linezolid (1 g BD) after two days. Following a positive signal from the automated BACTEC system indicating growth in both culture bottles on day 2, a sub-culture was done on MacConkey and 5% sheep blood agar and incubated at 37°C overnight. After 24 hours of incubation, small, circular, and smooth white colonies were observed on blood agar without any evidence of hemolysis (Figure 1). Gram stain showed gram-positive cocci and was identified as *L. lactis* cremoris by the Vitek2 compact system (bioMérieux, Marcy-l'Étoile, France).

The antibiotic susceptibility testing performed by Kirby-Bauer disk diffusion on Mueller-Hinton Agar demonstrated sensitivity to ceftriaxone (30 µg), doxycycline (30 µg), and ampicillin (10 µg), and resistance to levofloxacin (5 µg), erythromycin (15 µg), and clindamycin (2 µg), as per Clinical and Laboratory Standards Institute (CLSI) M45 [6].

Since the isolation of *Lactococcus* spp. in blood culture was unusual, a detailed history was taken from a family member to understand the source of the infection. It was reported that the patient had gone on a local pilgrimage, during which she was consuming outside food, dairy products, and water. Though unclear, the consumption of unpasteurized milk or dairy products could be the probable source of infection in this case. IV doxycycline (100 mg BD) was given for a period of seven days. On follow-up, repeat blood cultures were sent, which showed no growth after five days of incubation.

## Discussion

*L. lactis* species are gram-positive, lactic acid-producing bacteria, often used for the production of fermented dairy products and cheese [7]. Bacteria used in food preparation are commonly killed during digestion. However, *Lactococcus* remains viable even after transit through the gastrointestinal tract. This is thought to be the source of infection in humans [8]. Though commonly regarded as a non-pathogenic organism or contaminant, human infections have been reported. Hadjisymeou et al. [9] have observed 15 cases, including endocarditis, liver abscess, septicemia, pneumonitis, peritonitis, and brain abscess caused by *L. cremoris*. Of these, six cases have been associated with a history of exposure to unpasteurized dairy products. It has also been associated with the use of over-the-counter probiotic supplements [10].

The role of immune status as a risk factor for infection is debatable. Kaboré et al. report that immunocompromised patients were more susceptible to this infection [11]. However, in this case, the patient was immunocompetent. Similarly, Slaoui et al. reported that only 26% of the cases had a compromised immune status [4]. Slaoui et al. have observed that the incidence of *L. cremoris* infection was equal in men and women. It also affects all age groups, with the youngest patient being 19 months of age and the oldest being 79 years [4].

Although there are no standard guidelines for the treatment of *L. lactis* infection, multiple regimens have been considered based on antibiotic susceptibility test results, with penicillin or a third-generation cephalosporin being chosen most commonly. Gurley et al. treated *L. lactis* bacteremia patients with ertapenem and amoxicillin [10]. The patient recovered with this antibiotic regimen. In this case, since the patient did not show improvement with IV ceftriaxone, IV doxycycline was started based on the sensitivity report, to which the patient responded well. However, Kaboré et al. observed in their study that isolated strains of *L. lactis* subspecies *lactis* were multi-drug resistant. Owing to the irrational usage of antibiotics in animal husbandry, the sensitivity patterns for beta-lactams, particularly third-generation cephalosporins, have shown varied results [11]. The source of infection, risk factors, clinical presentation, and antibiotic sensitivity of this emerging pathogen are relatively sparse. Hence, documentation of such case scenarios is essential to gather more relevant information.

## Conclusions

Though *L. lactis* was formerly known to be nonpathogenic, it has been the etiological agent for several human infections. Although endocarditis, liver abscess, and endodontic infection by this organism have been observed in the past, an acute presentation of symptoms of food poisoning followed by bacteremia has rarely been reported. In a patient with a history of consumption of unpasteurized dairy products, it is essential for the clinician to rule out an *L. lactis* infection.
